# Surface Functionalization of Biomedical Ti-6Al-7Nb Alloy by Liquid Metal Dealloying

**DOI:** 10.3390/nano10081479

**Published:** 2020-07-28

**Authors:** Ilya Vladimirovich Okulov, Soo-Hyun Joo, Artem Vladimirovich Okulov, Alexey Sergeevich Volegov, Bérengère Luthringer, Regine Willumeit-Römer, Laichang Zhang, Lutz Mädler, Jürgen Eckert, Hidemi Kato

**Affiliations:** 1 Institute for Materials Research, Tohoku University, Katahira 2-1-1, Sendai 980-8577, Japan; jjsh83@imr.tohoku.ac.jp (S.-H.J.); hikato@imr.tohoku.ac.jp (H.K.); 2 University of Bremen, Badgasteiner Str. 1, 28359 Bremen, Germany; lmaedler@iwt.uni-bremen.de; 3 Leibniz Institute for Materials Engineering—IWT, Badgasteiner Str. 3, 28359 Bremen, Germany; 4 Institute of Natural Sciences and Mathematics, Ural Federal University, 620000 Ekaterinburg, Russia; alexey.volegov@urfu.ru; 5 Helmholtz-Zentrum Geesthacht, Institute of Materials Research, Division of Materials Mechanics, 21502 Geesthacht, Germany; okulovtema@yandex.ru; 6 Helmholtz-Zentrum Geesthacht, Institute of Material Research, Division of Metallic Biomaterials, 21502 Geesthacht, Germany; Berengere.Luthringer@hzg.de (B.L.); regine.willumeit@hzg.de (R.W.-R.); 7 School of Engineering, Edith Cowan University, 270 Joondalup Drive, Joondalup, Perth, WA 6027, Australia; l.zhang@ecu.edu.au; 8 Erich Schmid Instiute of Materials Physics, Austrian Academy of Sciences, Jahnstraße 12, 8700 Leoben, Austria; juergen.eckert@unileoben.ac.at; 9 Department of Materials Science, Chair of Materials Physics, Montanuniversität Leoben, Jahnstraße 12, 8700 Leoben, Austria

**Keywords:** surface functionalization, porous surface, biomaterial, dealloying, biocompatibility

## Abstract

Surface functionalization is an effective approach to change the surface properties of a material to achieve a specific goal such as improving the biocompatibility of the material. Here, the surface of the commercial biomedical Ti-6Al-7Nb alloy was functionalized through synthesizing of a porous surface layer by liquid metal dealloying (LMD). During LMD, the Ti-6Al-7Nb alloy is immersed in liquid magnesium (Mg) and both materials react with each other. Particularly, aluminum (Al) is selectively dissolved from the Ti-6Al-7Nb alloy into liquid Mg while titanium (Ti) and niobium (Nb) diffuse along the metal/liquid interface to form a porous structure. We demonstrate that the porous surface layer in the Ti-6Al-7Nb alloy can be successfully tailored by LMD. Furthermore, the concentration of harmful Al in this porous layer is reduced by about 48% (from 5.62 ± 0.11 wt.% to 2.95 ± 0.05 wt.%) after 30 min of dealloying at 1150 K. The properties of the porous layer (e.g., layer thickness) can be tuned by varying the dealloying conditions. In-vitro tests suggest improved bone formation on the functionalized porous surface of the Ti-6Al-7Nb alloy.

## 1. Introduction

Mechanical and biological compatibilities of metallic implant materials are required for their successful long-term application [1,2]. The mechanical compatibility of an implant material usually requires specific values of strength, elastic modulus, tensile ductility as well as fatigue and wear properties [2,3]. The required mechanical properties can be achieved, for example, via alloy design [4,5,6], tailoring of complex microstructures [7,8,9], or synthesis of porous [10,11] and composite materials [12,13]. The biological compatibility of implant materials can be achieved through avoiding toxic elements and surface functionalization (e.g., surface coating or surface roughening) [14,15,16,17]. Surface roughening increases implant/tissue bonding and enhances osteoinductivity due to increased interfacial contact between the implant and tissue [18]. This is particularly beneficial in clinical conditions of extreme bone atrophy [19]. Moreover, it was reported that osteoblasts on rougher surfaces exhibit a higher capacity to synthesize the bone matrix [20]. The surface roughness can be tailored by means of chemical or physical processes, including chemical etching [21] and dealloying [22,23].

There are two types of distinctly different dealloying approaches, namely, electrochemical dealloying [24] and liquid metal dealloying [25,26,27]. The approaches are rather complementary because each of these processes enables fabrication of a different spectrum of nanoporous materials that have been overlapping more and more in recent years. Particularly, electrochemical dealloying can be effectively used to fabricate mesoporous noble metals such as nanoporous gold [28] or platinum [29]. Recently, there have been some successful reports on fabrication of highly reactive non-noble mesoporous metals such as nanoporous aluminum [30]. In its turn, liquid metal dealloying has been used to fabricate a wide range of non-noble porous metals, including steels [31,32,33,34,35,36], titanium and titanium alloys [11,37,38,39], zirconium [12], tantalum [40], and cobalt-chromium [41], among others, as well as non-metallic porous materials such as silicon [42] and carbon [43,44]. Particularly important is the recent progress in the development of highly reactive mesoporous metals such as nanoporous magnesium [45] as well as multicomponent complex alloys such as nanoporous high-entropy alloys [46] by liquid metal dealloying.

In this work, liquid metal dealloying was successfully applied to synthesize the porous surface layer in the biomedical Ti-6Al-7Nb alloy and, thus, to functionalize its surface—the main objective of this study. The Ti-6Al-7Nb alloy samples with the functionalized (e.g., porous) surface demonstrate good cytocompatibility (the property of being harmless to cells—an indicator of biocompatibility) and improved bone formation which stimulates a better osseointegration, especially, in biomaterial graft sites [47]. The Ti-6Al-7Nb alloy is a two-phase alloy consisting of an alpha phase (hexagonal close-packed) stabilized by Al and beta phase (body centered cubic) stabilized by Nb [48,49]. The alpha phase is enriched in Al. Although the Ti-6Al-7Nb alloy is widely applied in medicine due to its excellent mechanical and biological properties [49], the release of harmful Al ions can lead to a cytotoxicity problem [50,51,52]. Therefore, the second objective of this work is reduction of the Al concentration in the surface layer of the Ti-6Al-7Nb alloy.

## 2. Materials and Methods

Bulk samples of Ti-6Al-7Nb (wt.%) were fabricated by means of sintering. The detailed synthesis route is described elsewhere [53]. Surface functionalization of Ti-6Al-7Nb was carried out by a two-stage process. First, the Ti-6Al-7Nb samples were dealloyed in a Mg metal melt (99.9%) at 1073 K for different times, namely, 10, 20, and 30 min. Upon dealloying, a modified surface layer consisting of Ti-rich and Mg-rich phases formed. Second, the Mg-rich phase was etched in 3 M aqueous solution of HNO_3_ for about 30 min in an ultrasonic bath followed by cleaning in deionized water and alcohol.

Structural investigations were performed by X-ray diffraction (XRD) in Bragg–Brentano geometry (Rigaku Ultima, Japan) with Cu-K_α_ radiation. The microstructure and chemical composition of the samples were explored by scanning electron microscopy (SEM, Karl Zeiss, Gemini Ultra 55, Germany) coupled with energy-dispersive X-ray (EDX) analysis (Bruker, Germany).

For biological tests, cells cultured on coverslips and on non-porous/non-dealloyed Ti-6Al-7Nb were selected as controls. For each assay, samples (cleaned and sterilized beforehand in 70% ethanol for 20 min in an ultrasonic bath) were placed into an agarose pre-coated 12-well plate. The surface of the investigated Ti-6Al-7Nb samples (size 1 × 6 × 15 mm^3^) was functionalized by dealloying at 1073 K for 30 min. The surface of the Ti-6Al-7Nb reference samples was in an as-cut condition (by a diamond saw). Afterwards, 5000 cells in 6 μL were added to each sample and incubated for 30 min to allow early cell adhesion. Then 3 mL of fresh medium were added to each well. Cells were further cultured for 3, 7, 14, and 21 days with medium change every 2–3 days.

Human umbilical cord perivascular cells (HUCPVCs) were obtained from umbilical cord samples. Written informed consent from the donor was obtained for the use of these samples in research and the study was conducted in accordance with the Declaration of Helsinki. HUCPVCs were isolated with the approval from the local ethical committee Ethik-Kommission der Ärztekammer Hamburg (Hamburg, Germany, PV4058), following the protocols from Sarugaser et al. [54]. The cord was cut into pieces of about 5 cm. The vessels were then isolated and tied together at the ends, leading to a vessel loop. Afterward, the loops were placed in a T-175 cell culture flask and cultured for 10 days in α-minimum essential medium (α-MEM; Fisher Scientific GmbH, Schwerte, Germany) and 15% fetal bovine serum (FBS; Biological Industries—NeoFroxx, Einhausen, Germany) and 1% antibiotics. After outgrowth of cells from the tissues, the medium was changed every 2–3 days.

Deoxyribonucleic acid (DNA) contents were measured (after 3, 7, 14, and 21 days culture; 3 biological and 3 technical replicates, respectively *n* = 9) to have an indication of the cell amount to see if the different materials had an influence on cell growth. DNA contents were also used as a normalization factor for alkaline phosphatase activity measurement. All chemicals were purchased from Sigma-Aldrich Chemie GmbH (Munich, Germany). Here, the cells were first digested overnight at 60 °C in a digestion solution containing 500 µL papain buffer solution (0.1 M NaH_2_PO_4_ and 5 mM EDTA, pH 6 in double distilled water—ddH_2_O), 5 µL β-mercaptoethanol, and 2.5 µL of papain solution (10 µg/mL papain in ddH2O). On the next day the samples were diluted in DNA dilution buffer (1:5; 2.5 M NaCl in 19 mM sodium citrate pH 7). Out of the latter solution 100 µL of diluted samples were pipetted in triplicate in a 96-well plate, and 50 µL of DNA working buffer (2 M NaCl in 15 mM sodium citrate pH 7) as well as 50 µL of bisbenzimide solution (2 µg/mL bisbenzimide in DNA working buffer) were incubated 15 min in the dark. The reactions were fluorometrically measured (excitation: 355 nm, emission: 460 nm) with a VICTOR3 multilabel plate reader (Perkin Elmer, MA, USA). Unknown DNA concentrations of samples were obtained by plotting the measured fluorescent emission to a DNA standard curve.

In vitro qualitative analysis of the cytotoxicity of the material was performed by using a LIVE/DEAD (Life Technologies, Darmstadt, Germany) assay. After 3, 7, 14, and 21 days culture, the staining solution was prepared by adding 4 μL Calcein AM (LIVE—green), and 10 μL ethidium homodimer-1 (DEAD—red) to 10 mL of phosphate-buffered saline (PBS). The samples were first washed with PBS to eliminate non-adherent cells, followed by immersion of each sample in 1.5 mL of staining solution, and incubated under cell culture conditions. The staining solution was then replaced by fresh α-MEM and samples were visualized by the fluorescent microscope (Nikon GmbH, Düsseldorf, Germany).

## 3. Results and Discussion

### 3.1. Structural Investigations

**Figure 1:**
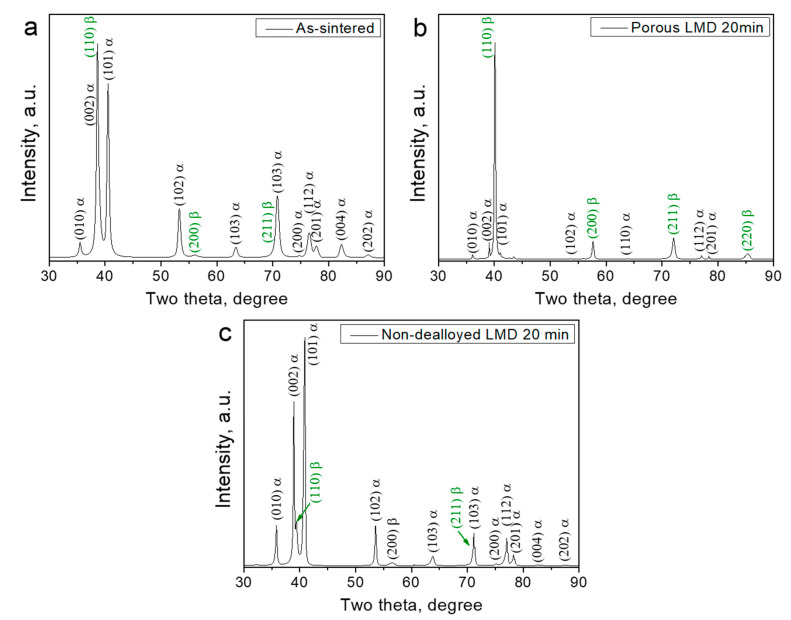
X-ray diffractograms of the as-sintered and dealloyed commercial Ti-6Al-7Nb alloy. (**a**) As-sintered; (**b**) Porous dealloyed layer of the Ti-6Al-7Nb alloy dealloyed for 20 min; (**c**) Non-dealloyed region of the Ti-6Al-7Nb alloy subjected to liquid metal dealloying for 20 min.

According to X-ray analysis, the as-sintered Ti-6Al-7Nb alloy possesses a dual-phase structure consisting of alpha titanium (hexagonal close-packed or hcp) and beta titanium (body-centered cubic or bcc) phases (Figure 1a). After dealloying, the porous layer of the Ti-6Al-7Nb alloy possesses a different structure as compared with the initial alloy and mainly consists of the beta and the remaining alpha titanium phases (Figure 1b). However, the phase structure of the undealloyed part of dealloyed Ti-6Al-7Nb remains unchanged. This consists of the alpha titanium and beta titanium phases, as can be seen in Figure 1c.

**Figure 2:**
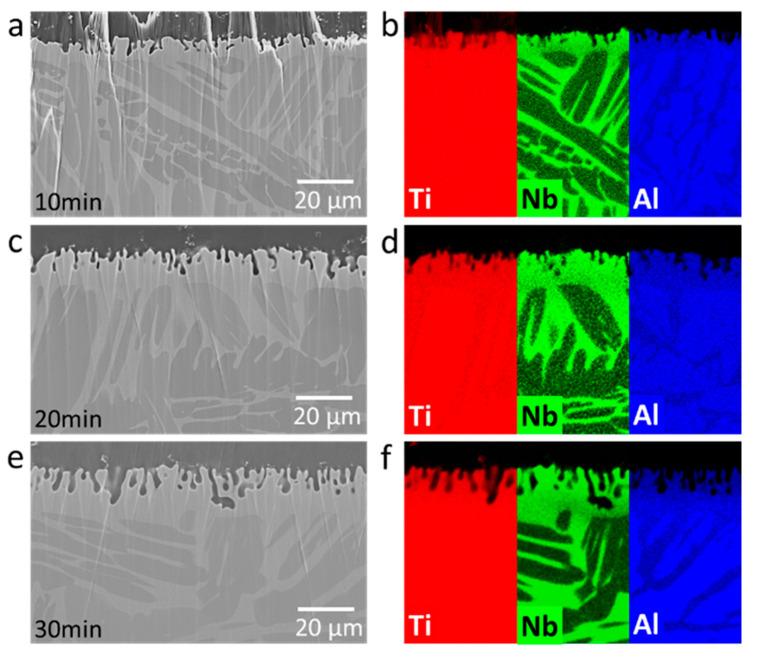
Scanning electron micrographs and corresponding element maps of an ion-milled cross section of Ti-6Al-7Nb dealloyed in liquid Mg at 1150 K for different times: (**a**,**b**) 10 min, (**c**,**d**) 20 min, and (**e**,**f**) 30 min.

**Figure 3:**
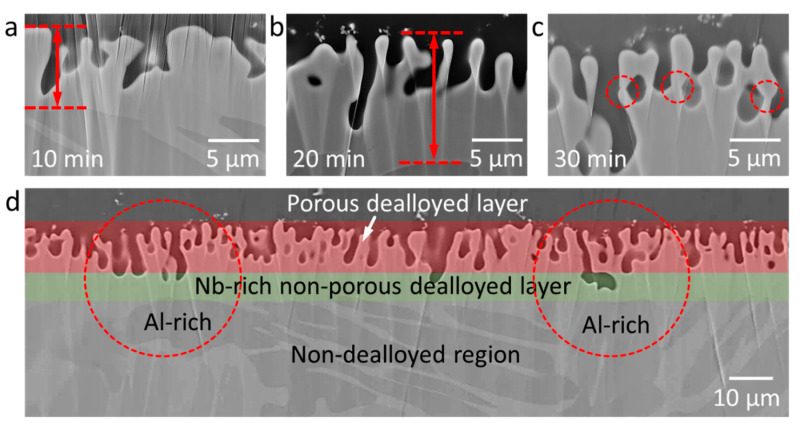
Scanning electron micrographs of the ion-milled cross section of Ti-6Al-7Nb dealloyed in liquid Mg at 1150 K for different times: (**a**) 10 min, (**b**) 20 min, and (**c**,**d**) 30 min. Note: The arrows in (**a**,**b**) indicate the thickness of the dealloyed layer. The dashed circles in (**c**) indicate joining of ligaments possessing a sintered-like neck morphology. The dashed circles in (**d**) indicate areas above the Al-rich phase regions possessing deeper pore depth compared to neighboring ones.

**Figure 4:**
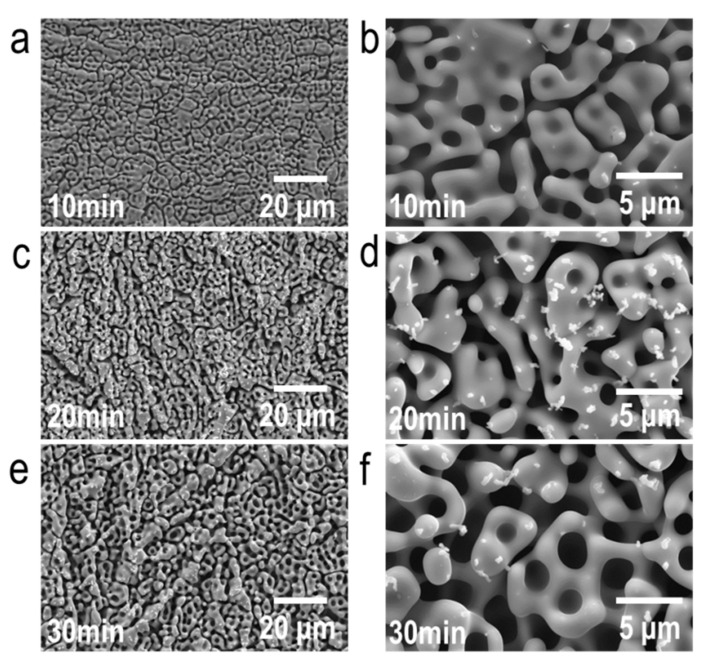
Scanning electron micrographs of the surface of Ti-6Al-7Nb dealloyed in liquid Mg at 1150 K for different time spans: (**a**,**b**) 10 min, (**c**,**d**) 20 min, and (**e**,**f**) 30 min. Note: The white spots in micrographs (**d**,**f**) are contamination particles.

**Figure 5:**
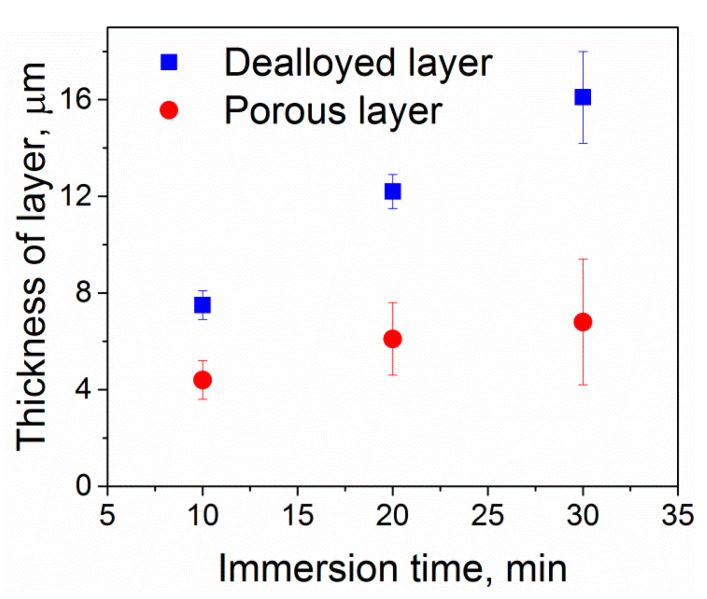
Surface characteristics of Ti-6Al-7Nb after dealloying. The thickness of the dealloyed (both porous and non-porous dealloyed layers, please see Figure 3) and the porous layers is plotted against the dealloying time. The error bars indicate standard deviation.

The microstructural analysis of the dealloyed samples reveals the formation of a dealloyed surface layer in each sample with a thickness of a few tens of micrometers. The dealloyed layer consists of porous and non-porous layers (Figure 2 and Figure 3). The porous dealloyed layer exhibits a bicontinuous morphology typical for liquid metal dealloying (Figure 4). As seen in the elemental maps (Figure 2), both dealloyed layers (e.g., porous and non-porous ones) are enriched in Nb and depleted in Al. The thickness of the whole dealloyed layer and the porous layer of the samples increases with increasing dealloying time (Figure 4 and Figure 5). The thickness of the dealloyed and the porous layers reaches 16.1 ± 1.9 μm and 6.8 ± 2.6 μm, respectively, after 30 min of dealloying (Figure 5).

The dealloying rate for the formation of the non-porous and the porous surface layers is not the same. Particularly, the thickness of the porous layer increases slower as compared with that of the whole non-porous layer. This is likely to be related with the parting limit [26] of the current alloy. The parting limit is a critical atomic percentage of the dissolving component (Al in this case) necessary for a structure to fully dealloy. The parting limit is different for different materials, but it often amounts to several tens of atomic percent (at.%). For example, in the case of Ti-Ta alloy system, about 40 at.% Ti is required to fully dealloy a Ti-Ta alloy in Cu melt [27]. The chemical composition of the current Ti-6Al-7Nb alloy (Table 1) is below the parting limit and, therefore, the formation of porous layer slows down with the dealloying time (Figure 5). The non-porous dealloyed layer depleted in Al evolves as a passivation layer preventing the further development of the porous layer. The high dealloying temperature supports diffusion of the dissolving element (Al) from the bulk material to the dealloying interface depleting the non-porous surface even further (Table 1).

As can be seen on the ion-milled cross section of the dealloyed samples (Figure 2 and Figure 3), the porous layer exhibits a channel-like morphology. The channel depth increases at higher dealloying times. The depth of these channels varies along the surface. Particularly, the deeper channels are observed in the Al-rich phase regions (Figure 3d). Since liquid metal dealloying is a diffusion process, these morphological features might be related with (i) different diffusion coefficient of Al in bcc and hcp titanium phases and (ii) different aluminum concentration gradients in the Al- and Nb-rich phases regions. The diffusion coefficient of Al in the hcp titanium phase for the similar titanium alloy Ti-6Al-4V is several orders of magnitude lower as compared with that found in the bcc titanium phase [55]. The lower diffusion coefficient of Al in the hcp titanium phase should lead to a slower dealloying rate of the hcp titanium (Al-rich phase) phase region. However, the opposite is observed. Thus, it might be that the effect of the Al concentration gradient prevails over the difference in diffusion coefficients of Al in the hcp and bcc phases. The aluminum concentration gradient is higher in the Al-rich phase regions (hcp phase) leading to a higher dealloying rate as compared with that in the Nb-rich phase regions (bcc phase). A sintering-like neck morphology of the ligament joints is observed for higher dealloying times (e.g., 30 min) (Figure 3c).

The microstructure of the non-dealloyed region (region directly below the dealloyed layer) of the samples subjected to dealloying is similar for all three dealloying conditions (Figure 2). The non-dealloyed region consists of two phases with brighter and darker contrasts, as shown in Figure 2 and Figure 3. The darker phase is Al-rich, and the brighter phase is Nb-rich according to EDX elemental analysis. The chemical compositions of the Al-rich and Nb-rich phases are Ti_89.2_Al_6.8_Nb_4.0_ and Ti_83.1_Al_4.8_Nb_12.1_ (wt. %), respectively. Since Nb stabilizes beta titanium and Al stabilizes alpha titanium phases [56], the Al-rich phase corresponds to alpha titanium and the Nb-rich phase is beta titanium. The phase structure of the non-dealloyed region of the Ti-6Al-7Nb alloy remains the same after dealloying, as can be seen from the X-ray diffraction patterns. This is probably related to the fact that the alloy was synthesized by sintering at elevated temperatures.

**Figure 6:**
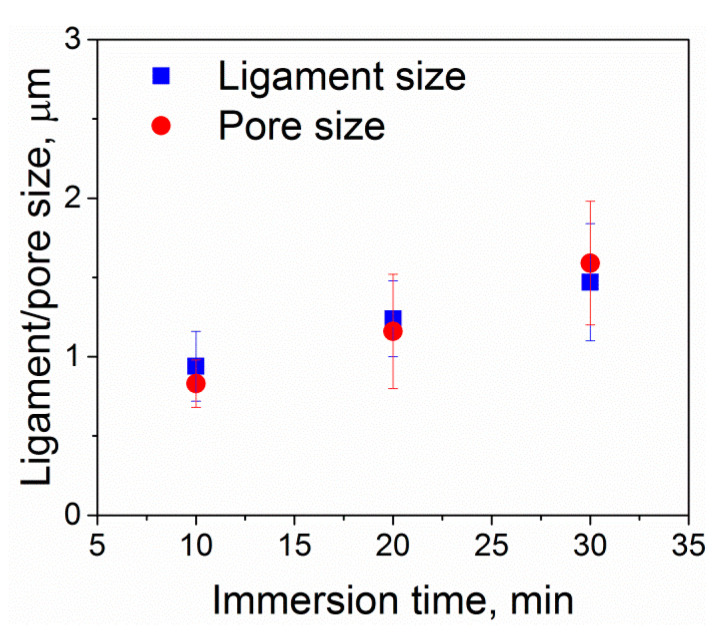
Surface characteristics of Ti-6Al-7Nb after dealloying in liquid Mg at 1150 K. Ligament/pore size platted against dealloying time. Note: The ligament/pore size was characterized by ImageJ software using the electron micrographs like those shown in Figure 4; the error bars indicate standard deviation.

In contrast to the non-dealloyed region (Figure 2), the porous surface layer of Ti-6Al-7Nb undergoes a significant microstructural evolution with the increasing dealloying time (the dealloying temperature was kept constant) (Figure 4). The porous surface layer represents a network of interconnected microscale ligaments. The ligament thickness increases from about 1 µm to 1.5 µm as the dealloying time increases from 10 to 30 min (Figure 4 and Figure 6). The same trend is observed for the pore size evolution. The pores exhibit nearly similar size as the ligaments (Figure 6).

**Figure 7:**
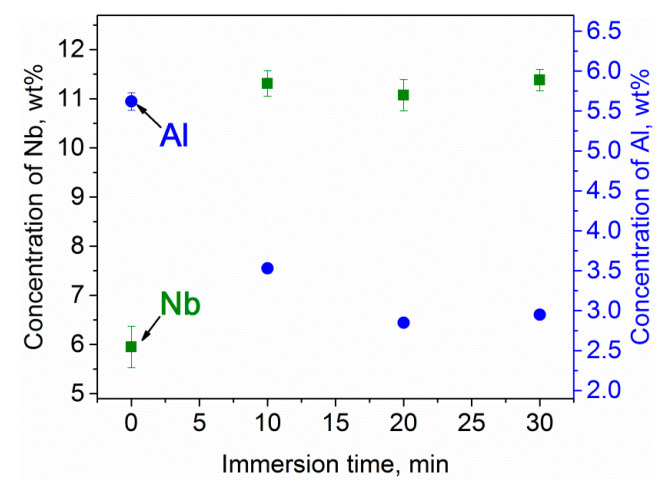
Concentration of Nb and Al in the surface layer of Ti-6Al-7Nb before and after dealloying (porous layer) in liquid Mg at 1150 K for different times. Note: The size of the error bars in the case of Al is below that of the round symbols; the error bars indicate standard deviation.

Upon dealloying, the average chemical composition of the porous surface layer changes as compared with the initial chemical composition of the Ti-6Al-7Nb alloy. The chemical composition of the porous surface layer strongly depends on the dealloying conditions (Figure 7 and Table 1). Particularly, the average concentration of harmful Al decreases from 5.62 ± 0.11 wt.% (parent alloy) to 2.95 ± 0.05 wt.% (30 min of dealloying). In turn, the concentration of Nb increases from about 5.95 ± 0.42 wt.% (parent alloy) to 11.38 ± 0.22 wt.% (30 min of dealloying), as shown in Figure 7. The dissolution of Al from the surface layer is thermodynamically favorable due to the negative enthalpy of mixing between Al and Mg [57]. On the contrary, the enthalpies of mixing between Ti and Mg as well as Nb and Mg are positive [57]. Therefore, the Mg melt rejects both Ti and Nb elements.

### 3.2. Cytocompatibility Tests

**Figure 8:**
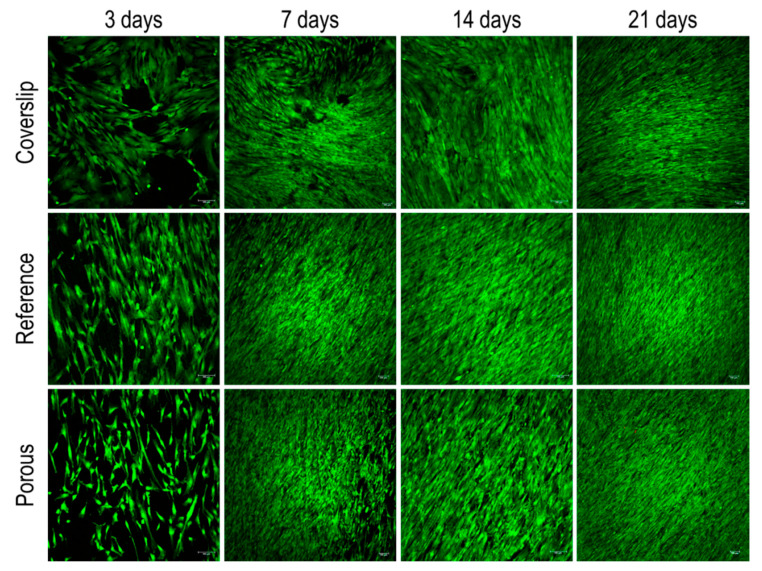
Representative fluorescence images of human umbilical cord perivascular cells (HUCPVCs) cultured on porous (dealloyed) and reference (non-dealloyed) samples. Fluorescence LIVE (green)/DEAD (red) staining was performed after 3, 7, 14, and 21 days of cell culturing. For interpretation of the references to color in this figure legend, the reader is referred to the web version of this article.

To ensure the cytocompatibility of the Ti-6Al-7Nb alloy with the porous layer obtained by liquid metal dealloying, a LIVE/DEAD staining was performed after 3, 7, 14, and 21 days. The non-processed Ti-6Al-7Nb alloy samples (without porous layer) were selected as a comparison in order to interpret the results. Figure 8 presents staining micrographs of the dealloyed and non-dealloyed (reference) Ti-6Al-7Nb samples. The dealloyed samples exhibit good cytocompatibility similar to that observed for non-dealloyed ones.

**Figure 9:**
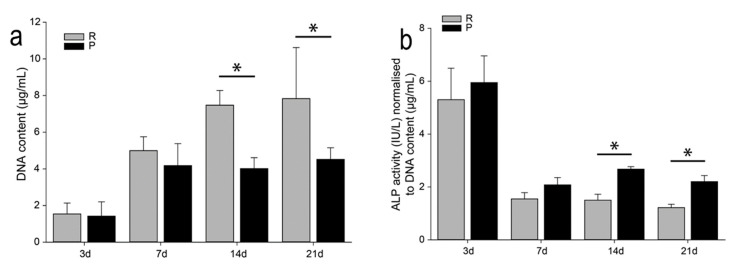
(**a**) DNA content and (**b**) ALP activity of porous and non-porous Ti-6Al-7Nb samples after 3, 7, 14, and 21 days of culturing (grey columns—reference (R), non-porous/non/dealloyed and black columns—porous (P) Ti-6Al-7Nb samples). Stars indicate significant differences between two groups (one-way RM ANOVA; * *p* < 0.05).

The DNA (equivalent to the cell number) content was measured for the quantitative comparison of samples (Figure 9a). As can be seen from the DNA content estimate, less cells are present on the porous samples compared to the non-porous ones. However, at days 14 and 21, the ALP activity (Figure 9b) is significantly upregulated for the porous samples. The ALP activity is a useful biochemical marker for bone formation, especially mineralization [58]. Furthermore, differentiation progression usually coincides with proliferation decrease [59]. Therefore, the porous surface of dealloyed Ti-6Al-7Nb samples may be more favorable for cell differentiation than the surface of non-dealloyed material. This might even stimulate better osseointegration of the developed porous materials which is quite advantageous in view of possible application as implant material for musculoskeletal application.

## 4. Conclusions

We successfully synthesized the porous surface of the commercial biomedical Ti-6Al-7Nb alloy by liquid metal dealloying (LMD). It was demonstrated that pore size, shape of pores, and the depth of the porous layer can be effectively tuned by controlling the dealloying parameters. Importantly, the concentration of harmful Al in the porous layer was significantly (up to 48%) reduced. Samples with a functionalized surface (e.g., porous surface layer) demonstrated good cytocompatibility and improved bone formation when compared with the reference (non-porous) samples. The current findings demonstrate opportunities of liquid metal dealloying for the surface functionalization of biomedical alloys including synthesis of porous surface layers as well as tailoring of the chemical composition of these porous layers. The LMD-tailored porous surfaces might also be used for drug delivery.

## Figures and Tables

**Table 1:** Chemical composition of the surface before and after dealloying (porous layer) at 1150 K.

Dealloying Time (min)	Ti (wt.%)	Nb (wt.%)	Al (wt.%)
0 (initial chemical composition)	88.43 ± 0.08	5.95 ± 0.42	5.62 ± 0.11
10	85.16 ± 0.21	11.31 ± 0.26	3.53 ± 0.06
20	86.08 ± 0.17	11.07 ± 0.32	2.85 ± 0.04
30	85.67 ± 0.19	11.38 ± 0.22	2.95 ± 0.05
